# Examining the independent and interactive association of physical activity and sedentary behaviour with frailty in Chinese community-dwelling older adults

**DOI:** 10.1186/s12889-022-13842-1

**Published:** 2022-07-26

**Authors:** Na Li, Feng Huang, Hong Li, Siyang Lin, Yin Yuan, Pengli Zhu

**Affiliations:** 1 The Shengli Clinical Medical College of Fujian Medical University, 134 Dongjie Road, Fuzhou, Fujian 350100 People’s Republic of China; 2 Department of Nursing, Fujian Provincial Hospital, Fuzhou, Fujian People’s Republic of China; 3 The School of Nursing, Fujian Medical University, Fuzhou, Fujian People’s Republic of China; 4 Fujian Provincial Key Laboratory of Geriatrics, 134 Dongjie Road, Fuzhou, Fujian 350100 People’s Republic of China; 5 Department of Geriatric Medicine, Fujian Provincial Hospital, 134 Dongjie Road, Fuzhou, Fujian 350100 People’s Republic of China

**Keywords:** Physical activity, Sitting time, Frailty, Interaction, Dose-response

## Abstract

**Background:**

While physical inactivity or prolonged sitting has been linked to an increased risk of frailty, the interaction between sitting time (ST), physical activity (PA) and frailty is not well understood. The aim of this study was to examine the dose-response relationship between PA, ST and frailty and further to evaluate the interaction effect of PA and ST on frailty in the context of regular COVID-19 epidemic prevention and control in China.

**Methods:**

A cross-sectional analysis was performed on 1458 participants (age ≥ 60) enrolled from a prospective cohort study of frailty in elderly people of Fujian Province. PA and ST levels were assessed using the International Physical Activity Questionnaire. A 40-item frailty index (FI) quantified frailty. Multivariable logistic regression and linear regression models were applied to examine the dose-response relationship between PA or ST and frailty level. Interaction plots were used to visualise the interaction effects of PA and ST on frailty.

**Results:**

Compared with light PA, the odds ratios (ORs) for frailty were significantly lower for moderate PA (OR, 0.609 [95% CI, 0.419, 0.885], *P* < .001) and vigorous PA (OR, 0.399 [95% CI, 0.236,0.673], *P* < .001). Comparing subjects with ST <  4 h/day, those with ST ≥ 8 h/day were significantly more likely to be diagnosed with frailty (OR, 3.140 [95% CI, 1.932, 5.106], *P* < .001), 6–8 h/day (OR, 1.289 [95% CI, 0.835, 1.989], *P* >0.05), and 4–6 h/day (OR, 1.400 [95% CI, 0.972, 2.018], *P* >0.05). Each one unit increase in metabolic equivalents (h/day) of PA was related to an average 0.928 (0.887, 0.971) decrease in prevalence of frailty, while each one unit increase in sitting time (h/day) was related to average 1.114 (1.046,1.185) increase in prevalence of frailty. Negative interactive effects of PA and ST on frailty were observed (*P* < 0.001).

**Conclusion:**

There are nonlinear and linear dose-response relationships between PA, SB and frailty respectively. In addition, excess ST may counteract the beneficial effects of PA on frailty. Interventions that focus on reducing excess ST may be effective strategies to reduce the risk of frailty and should be taken seriously by public health authorities, especially in the context of regular epidemic prevention and control in China.

**Supplementary Information:**

The online version contains supplementary material available at 10.1186/s12889-022-13842-1.

## Background

Ageing is accelerating at an unprecedented rate all over the world, China has the largest and fastest ageing population in the world [1]. Rising population life expectancy will inevitably leads to an increase in the occurrence of chronic diseases and disabilities [2]. Although ageing can lead to disability, in fact, elderly people of the same biological age can have completely different health states [3]. The term of frailty is used to explain this heterogeneity in ageing [4].

Frailty is a complex concept characterized by an increase in vulnerability of the body and decrease in the ability to resist stressors [5]. Frailty is a strong predictor of multiple adverse outcomes, including falls, hospitalization, disability and premature mortality [6, 7]. Among community-dwelling elder adults in China, the prevalence of frailty was on average 12%, resulting in heavy economic, political and social burdens on families and countries [8]. Reducing risk factors and increasing protective factors, particularly modifiable lifestyle behaviors, can play an important role in developing prevention strategies to manage frailty [9].

There are a variety of protective factors to prevent frailty, and physical activity-based approaches such as physical activity (PA) and sedentary behaviors (SB) may be the most effective strategies for slowing the progression of frailty [10, 11]. However, older adults are the most sedentary and least physically active age group, epidemiological evidence suggests that more than 85% of older adults do not meet the World Health Organization physical activity recommendation of 150 minutes of moderate to vigorous physical activity (MVPA) per week [12]. On the contrary, they spend about 9.4 h/day in sedentary behaviors such as sitting [13]. A qualitative research also found older adults often interpreted sedentary behavior as synonymous with a lack of MVPA, and many perceived the word ‘sedentary’ as having negative connotations and are unwilling to identify themselves as sedentary [14].

Over the past decade, there has been a large body of previous research focusing on the effects of increased physical activity or decreased sedentary time on frailty [15–17] and emerging evidence has shown a dose-response relationship between PA or SB with frailty levels [18, 19], Howerver, the interaction among PA, ST and frailty is not well understood [20]. Whether PA can counteract the adverse effects of ST remains to be clarified. Understanding how these two adjustable risk factors are combined in frailty may be crucial in developing quantitative guidelines to limit the amount of time spent sitting by frail older adults.

In addition, under the circumstance of regular COVID-19 epidemic prevention and control in China over the past 2 years, people are more confined to their homes, and the elderly in the community tend to sit more and move less [21]. In view of this, it was necessary to conduct this study to explore the dose-response relationship between PA, ST and frailty in community-dwelling Chinese older adults and further to evaluate the interaction effect of PA and ST with frailty.

## Material and methods

### Study design and study population

This cross-sectional study was conducted in Fujian Province, China, as the preliminary phase of the project “Prospective cohort study of frailty in elderly people of Fujian Province”, which aims to explore the influence of ageing and frailty in the elderly for clinical decision making in frailty risk assessment. From July to December 2021, the eligible elderly population in Fuzhou Community Health Service Center of Fujian Province was recruited by telephone calls and posters. Inclusion criteria were men and women over the age of 60, informed consent and volunteered to participate in the study and ability to complete scale evaluation and physical examination. Exclusion criteria were life expectancy < 6 months because of critical disease or advanced tumour; long-time bedridden, completely disabled; severe visual, hearing or speech impairment. The study was in accordance with the 1975 Declaration of Helsinki and approved by the ethics committee of FuJian Provincial Hospital.

### Sample

Meta-analysis suggests that the prevalence of frailty in the non-hospitalised elderly population aged 60 and above in China is 12% [8]. The sample size of the cross-sectional study was calculated by $n=\frac{z_{\sigma}^2\times pq}{d^2}$.

where *Z*_σ_ is the significance test statistic, α = 0.05, *Z*_σ_ = 1.96, p is the estimated frailty incidence rate of 12%, q = 1-p; *d* is the allowable error, in this study 0.02; the minimum sample size calculated is 1063. Considering a projected 20% sample loss because of questionnaire quality, the minimum sample size required was 1276.

A total of 1508 participants over the age of 60 were recruited from Community Health Service Center of Fujian Province. After excluded participants who have extreme SB and PA values (*n* = 14) and missing data on frailty index (Grip Strength, Balance, Fatigue) (*n* = 29) or potential confounders (*n* = 7), a total of 1458 subjects were included in the final analysis. The flowchart of participant selection for this study analysis was provided in Fig. S1.

### Measurements

#### PA assessment

The short from of the International Physical Activity Questionnaire (IPAQ), which has been validated in China, was used to assess physical activity (PA) level [22].

The IPAQ-SF consists of seven items and provides information on the time spent in vigorous-intensity activity (eg, jogging, swimming, running), moderate-intensity activity (eg, dancing, riding a bike, cleaning house) and walking. The IPAQ-SF required the subjects to recall the number of days they performed each activity (frequency) and the length of time (duration) they were involved in each daily activity in the last 7 days. The formula of IPAQ was as follows: the total physical activity (MET/min/w) = the MET (metabolic equivalents) value of physical activity × the amount of time spent on physical activity per day (min/d) × the number of days of physical activity per week (d/w). MET values for vigorous-intensity activity, moderate-intensity activity, and walking were 8, 4, and 3.3, respectively. We converted the continuous variables corresponding to the total physical activity into three categorical variables, which uses cut-off values of 600 and 3000 MET min/w as follows: low total physical activity (< 600 MET/min/w), moderate total physical activity (600–3000 MET/min/w) and high total physical activity (≥ 3000 MET/min/w) [23].

#### Sedentary behaviour assessment

The researchers assessed ST by asking “How many h in a 24-h day do you typically spend sitting”? This includes working at a desk or computer, visiting friends, riding in a car, reading, playing cards or watching TV but does not include sleeping time. The average amount of time spent sitting per day over the past 7 days fell into four categories, 4 h/d, 4 ~ 6 h/d, 6 ~ 8 h/d and ≥ 8 h/d, similar to the classification used in recent studies [24].

### Frailty measure

The frailty index (FI), which is based on the theory of health defects, was used to measure the degree of frailty [25]. The FI refers to the proportion of potential unhealthy measurement indicators of an individual among all measurement indicators at a certain time point. The more defects a person has, the more likely he or she is to be in a frail state. In the present study, the FI consisted of 40 variables (see Table S1 for variables), including multi-dimensional indicators such as medical signs, medical diagnosis, activities of daily living and performance tests (walking speed, grip strength and TUG) [26]. According to previous research, FI 0.2 was defined as the threshold for entering the frailty state, and individuals were divided into non-frailty (< 0.2) and frailty (0.2–1.0) groups [27].

### Covariates

Baseline data were collected by trained researchers through face-to-face interviews using standardized questionnaires. Main contents includes general demographic information (age and gender), socioeconomic attributes (marital status, living status, education level, now or before retirement occupation, average monthly income, method for medical payments), lifestyle (smoking status, alcohol consumption, etc.) and history of disease and medication. The education level was divided into primary school and below, middle school, high school, college and master’s degree and above; Marital status is classified as married, widowed, divorced or other. The average monthly income was divided into <3000 RMB, 3000–6000 RMB, 6000–10,000 RMB and >10,000 RMB. Occupations were classified as civil servants or professional technicians in state units, workers, commercial, service or freelance workers, manual or unemployed. Living status was divided into living with family, living with others and living alone. Smoking and drinking status were divided into current, former and never groups. Weight and height, waist circumference, blood pressure and BMI were measured and calculated using standard methods.

### Statistical analysis

SAS 9.4 (Cary, NC) was used to analyse the data, and the measurement data were expressed as mean ± standard deviation, and the *t-*test was used to compare the two groups. The *×* ^*2*^ test was used to compare the two groups of categorical data. If the theoretical frequency was too small, Fisher’s exact probability method was used.

A multiple linear regression model was used to analyse the relationship between PA or ST and FI, expressed as *β* values of 95% confidence intervals (CI), with light physical activity and minimum sitting time (< 4 h/day) as reference categories, respectively. Multivariate adjusted logistic regression models were also used to assess the association between PA or ST and the prevalence of frailty, with results expressed as odds ratios (OR) with corresponding 95% CI. Two models were adopted to assess association of PA, ST and frailty. Model 1 was adjusted according to PA and ST levels; Model 2 adjusted for age, gender, education level, marital status, average monthly income, smoking status, drinking status, BMI, ST and PA.

A cross-product term was added to the logistic regression model to evaluate the statistical significance of the interaction between PA and ST on frailty. A restricted cubic spline regression was used to investigate the dose-response relationship between continuous PA-MET-h/day or ST (h/day) and frailty.

We conducted joint analysis of sitting time, physical activity and frailty, comparing groups with different amounts of sitting time and physical activity with the combined vigorous PA and lowest ST (< 4 h/day) groups serving as the reference group.

A generalized linear model was used to visualize the interaction of ST (h/day) and PA (MET-h/day) on frailty. In the interaction diagram, the effect of MET-h/day is estimated with 95% CI as a function of the increase in ST (h/day).

## Results

### Descriptive statistics

A total of 1458 subjects completed questionnaires and physical examination. The Table 1 shows the demographic characteristics of the study participants by frailty status. The participants’ mean age was 72.38 years (SD = 7.28 years) and 59.88% were females. The mean FI was 0.14 (SD = 0.07). In the previous week, the non-frailty group had higher MET-h/d (5.24 ± 4.35 vs 3.59 ± 3.45) and lower sedentary time (4.51 ± 2.22 h/day vs 5.16 ± 2.52 h/day) than the frailty group. Student’s *t*-test and *×* ^*2*^ test results showed that age, educational level, living status, marital status, economic income, drinking status, comorbidity, polypharmacy, PA and sedentary time had statistically significant differences between participants according to frailty status.

**Table 1:** Distribution of demographic, behavioural, and health status of study participants by frailty status (*n* = 1458)

Characteristics	Total (*n* = 1458)	Non-frail (*n* = 1163)	Frail (*n* = 295)	*t/x*^*2*^	*P* value
Age (year, mean ± SD)	72.38 ± 7.28	71.37 ± 6.68	76.37 ± 8.16	−9.040	< 0.0001
Gender (*n,* %)				1.734	0.188
Male	585 (40.12)	477 (41.00)	108 (36.47)		
Female	873 (59.88)	686 (59.00)	187 (63.53)		
Education level (*n,* %)				17.215	0.002
Primary school and below	270 (18.49)	192 (16.52)	78 (26.27)		
Middle school	383 (26.27)	300 (25.77)	83 (28.24)		
High school	474 (32.54)	398 (34.23)	76 (25.88)		
College	328 (22.46)	270 (23.18)	58 (19.61)		
Master’s degree and above	3 (0.24)	3 (0.30)	0 (0.00)		
Living status (*n*, %)				75.185	< 0.0001
Living with family	1225 (84.05)	1024 (88.05)	201 (68.14)		
Living with others	138 (9.44)	69 (5.97)	69 (23.39)		
Living alone	95 (6.51)	70 (5.98)	25 (8.47)		
Marital status (*n*, %)				34.620	< 0.0001
Married	1132 (77.62)	943 (81.08)	189 (64.07)		
Widowed	303 (20.79)	204 (17.54)	99 (33.56)		
Divorced or other	23 (1.59)	16 (1.38)	7 (2.37)		
Occupations (*n*, %)				0.668	0.716
Civil servants or professional technicians in state units	776 (53.25)	626 (53.83)	150 (50.98)		
Workers, commercial, service or freelance workers	552 (37.86)	435 (37.41)	117 (39.61)		
Manual or unemployed	130 (8.89)	102 (8.76)	28 (9.41)		
Average monthly income (*n*, %)				11.906	0.008
<3000 RMB	485 (33.25)	394 (33.88)	91 (30.85)		
3000 ~ 6000 RMB	826 (56.67)	657 (56.49)	169 (57.29)		
6000 ~ 10,000 RMB	139 (9.52)	110 (9.46)	29 (9.83)		
>10,000 RMB	8 (0.56)	2 (0.17)	6 (2.03)		
Main medical payment methods (*n*, %)				0.263	0.608
All at one’s own expense	47 (3.25)	39 (3.38)	8 (2.75)		
Partial or full payment of medical insurance	1411 (96.75)	1124 (96.62)	287 (97.25)		
Smoking status (*n*, %)				0.166	0.920
Never	1238 (84.92)	988 (84.98)	250 (84.71)		
Current	112 (7.70)	88 (7.56)	24 (8.24)		
Former	108 (7.38)	87 (7.46)	21 (7.06)		
Drinking status (*n,* %)				6.715	0.035
Never	1259 (86.35)	993 (85.38)	266 (90.17)		
Current	149 (10.24)	132 (11.35)	17 (5.76)		
Former	50 (3.41)	38 (3.27)	12 (4.07)		
Comorbidity (*n*, mean ± SD)	2.23 ± 1.79	1.99 ± 1.63	3.18 ± 2.05	−8.590	< 0.0001
Polypharmacy (*n*, mean ± SD)	3.27 ± 2.88	2.80 ± 2.62	5.11 ± 3.11	−10.910	< 0.0001
Physical activity (*n*, %)				38.016	< 0.0001
Light	235 (16.11)	154 (13.23)	81 (27.45)		
Moderate	922 (63.25)	743 (63.88)	179 (60.78)		
Vigorous	301 (20.63)	266 (22.89)	35 (11.76)		
MET-h/day (mean ± SD)	4.91 ± 4.24	5.24 ± 4.35	3.59 ± 3.45	6.480	< 0.0001
Sitting time (*n*, %)				31.719	< 0.0001
< 4 h/day	583 (40.00)	499 (42.89)	84 (28.63)		
4–6 h/day	467 (32.06)	361 (31.04)	106 (36.08)		
6–8 h/day	272 (18.65)	218 (18.71)	54 (18.43)		
≥ 8 h/day	136 (9.29)	86 (7.36)	50 (16.86)		
Sitting time (h/day, mean ± SD)	4.64 ± 2.30	4.51 ± 2.22	5.16 ± 2.52	−3.780	< 0.0001
BMI (kg/m^2^, mean ± SD)	24.60 ± 3.16	24.62 ± 3.07	24.51 ± 3.49	0.490	0.626
Frailty index (mean ± SD)	0.14 ± 0.07	0.11 ± 0.04	0.25 ± 0.06	−37.310	< 0.0001

### Associations of PA or ST with FI and frailty

The results of multiple linear regression showed that after adjusting for other factors, in model 2 the *βs* and 95% CIs for moderate and vigorous PA were − 0.024(− 0.035,-0.013) and − 0.034 (− 0.047, − 0.021), respectively compared with light PA. The *βs* and 95% CIs for ST ≥ 8 h/day, 6–8 h/day and 4–6 h/day were 0.034 (0.020, 0.048), 0.011 (0.001, 0.022) and 0.013 (0.003, 0.022), respectively compared with < 4 h/day ST. Each one unit increase in MET (h/day) of PA was related to an average 0.0021(−.0030, −.0012) decrease in FI, while each one unit increase in ST (h/day) was related to an average 0.0034 (0.0017, 0.0051) increase in the FI, as shown in Table 2.

**Table 2:** Association of physical activity and daily sitting time with the frailty index (FI)

	Number	FI (mean ± SD)	*Β* (95% CI)			
			Model 1	*P*	Model 2	*P*
Physical activity						
Light	235	0.17 ± 0.09	0(Ref.)		0(Ref.)	
Moderate	922	0.13 ± 0.07	−0.033(−0.044,-0.022)	< 0.0001	−0.024(−0.035,-0.013)	< 0.0001
Vigorous	301	0.12 ± 0.06	−0.050(− 0.063,-0.037)	< 0.0001	−0.034(−0.047,-0.021)	< 0.0001
MET-h/day			−0.0033(− 0.0042,-0.0023)	< 0.0001	− 0.0021(− 0.0030,-0.0012)	< 0.0001
*P* value for trend			< 0.0001		< 0.0001	
Daily Sitting time						
< 4 h/day	583	0.12 ± 0.06	0(Ref.)		0(Ref.)	
4–6 h/day	467	0.14 ± 0.07	0.017 (0.007,0.026)	< 0.0001	0.013 (0.003,0.022)	0.007
6–8 h/day	272	0.14 ± 0.08	0.014 (0.002,0.025)	< 0.0001	0.011 (0.001,0.022)	0.039
≥ 8 h/day	136	0.16 ± 0.09	0.039 (0.024,0.053)	< 0.0001	0.034 (0.020,0.048)	< 0.0001
Per h increased			0.0038 (0.0020,0.0055)	< 0.0001	0.0034 (0.0017,0.0051)	0.000
*P* value for trend			< 0.0001		< 0.0001	

Multivariable model 1 adjusted for PA level and ST; model 2 adjusted for educational level, living status, marital status, economic income, drinking status, comorbidity, and polypharmacy

*SD* Standard deviation, *CI* Confidence interval

Multivariate logistic regression showed that, on the premise of adjusting the influence of other factors, in modal 2, compared with light PA, the ORs for frailty were significantly lower than for moderate PA (OR, 0.609 [95% CI, 0.419, 0.885]) and vigorous PA (OR, 0.399 [95% CI, 0.236, 0.673]). While compared with patients with ST <  4 h/day, those with ST ≥ 8 h/day were significantly more likely to be diagnosed as frail (OR, 3.140 [95% CI, 1.932, 5.106]), 6–8 h/day (OR, 1.289 [95% CI, 0.835, 1.989]), and 4-6 h/day (OR, 1.400 [95% CI, 0.972, 2.018]). Each one-unit increase in MET (h/day) of PA was related to an average 0.928 (0.887, 0.971) decrease in prevalence of frailty, while each one- unit increase in ST (h/day) was related to average1.114 (1.046, 1.185) increase in prevalence of frailty, as shown in Table 3.

**Table 3:** Association of physical activity (PA) and daily sitting time (ST) with prevalence of frailty

	Cases/number	Prevalence (95% CI)	OR (95% CI)			
			Model 1	*P*	Model 2	*P*
Physical activity						
Light	81/235	34.48 (28.28, 41.26)	1(Ref.)		1(Ref.)	
Moderate	179/922	19.45 (16.85, 22.34)	0.468 (0.332,0.659)	< 0.0001	0.609 (0.419,0.885)	0.009
Vigorous	35/301	11.54 (8.16, 16.03)	0.244 (0.150,0.396)	< 0.0001	0.399 (0.236,0.673)	0.001
MET-h/day			0.883 (0.845,0.923)	< 0.0001	0.928 (0.887,0.971)	0.001
*P* value for trend			< 0.0001		0.002	
Daily sitting time						
< 4 h/day	85/583	14.48 (11.67,17.84)	1(Ref.)		1(Ref.)	
4–6 h/day	106/467	22.77 (18.94,27.11)	1.690 (1.197,2.385)	0.003	1.400 (0.972,2.018)	0.071
6–8 h/day	54/272	20.00 (15.36,25.60)	1.402 (0.930,2.113)	0.107	1.289 (0.835,1.989)	0.252
≥ 8 h/day	50/136	36.75 (28.56,45.79)	3.509 (2.215,5.560)	< 0.0001	3.140 (1.932,5.106)	< 0.0001
Per h increased			1.121 (1.057,1.189)	< 0.0001	1.114 (1.046,1.185)	0.001
*P* value for trend			< 0.0001		< 0.0001	
*P* ST × Physical activity			0.013		0.036	

Multivariable model 1 adjusted for PA level and ST; model 2 adjusted for educational level, living status, marital status, economic income, drinking status, comorbidity, polypharmacy

*OR* Odds ratio, *CI* Confidence interval

**Fig. 1:**
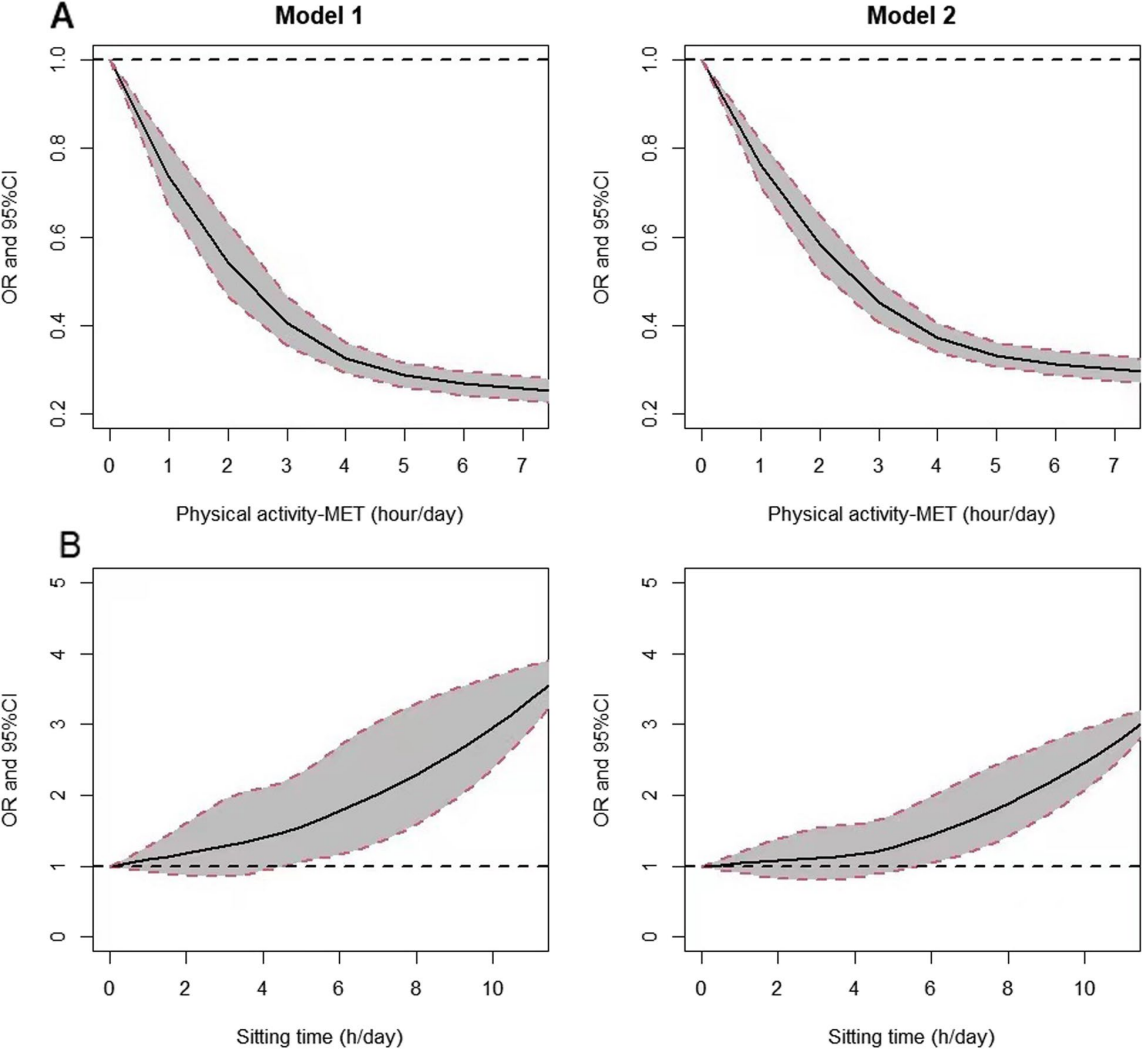
The association between continuous PA-MET (h/day) or ST (h/day) and frailty. Association of physical activity (PA) (MET-h/day) (**A**) or sitting time (ST) (h/day) (**B**) on risk of frailty were analysed by using restricted cubic splines. Model 1: Adjusted for PA level and ST; Model 2: Adjusted for educational level, living status, marital status, economic income, drinking status, comorbidity and polypharmacy

The dose-response relationship between continuous PA-MET (h/day) or ST (h/day) with frailty was investigated by restricted cubic spline regression, as shown in Fig. 1.

### Joint analysis of PA and ST effects on frailty

The joint analysis, as shown in Table 4, indicated that an association between ST and increased frailty was observed only among lightly physically active older adults but not among highly physically active adults (except for the sedentary > 8 h group). Notably, those who spent the most time sitting (> 8 h/day) had a 8-fold increased risk of frailty.

**Table 4:** Joint effects on frailty by different combinations of physical activity level and sitting time

	Sitting time	OR (95%CI)	*P*
Vigorous physical activity			
	< 4 h/d	1 (Ref.)	
	4-6 h/d	1.840 (0.642,5.278)	0.257
	6-8 h/d	3.139 (0.961,10.253)	0.058
	≥ 8 h/d	5.228 (1.644,16.628)	0.005
Moderate physical activity			
	< 4 h/d	2.129 (0.918,4.942)	0.078
	4-6 h/d	2.935 (1.264,6.814)	0.012
	6-8 h/d	2.964 (1.226,7.164)	0.016
	≥ 8 h/d	6.498 (2.525,16.720)	< 0.0001
Light physical activity			
	< 4 h/d	4.432 (1.706,11.513)	0.002
	4-6 h/d	5.407 (2.090,13.986)	0.001
	6-8 h/d	2.693 (0.899,8.068)	0.077
	≥ 8 h/d	8.223 (3.073,28.012)	< 0.0001

### Interactive effects of PA and ST on frailty

**Fig. 2:**
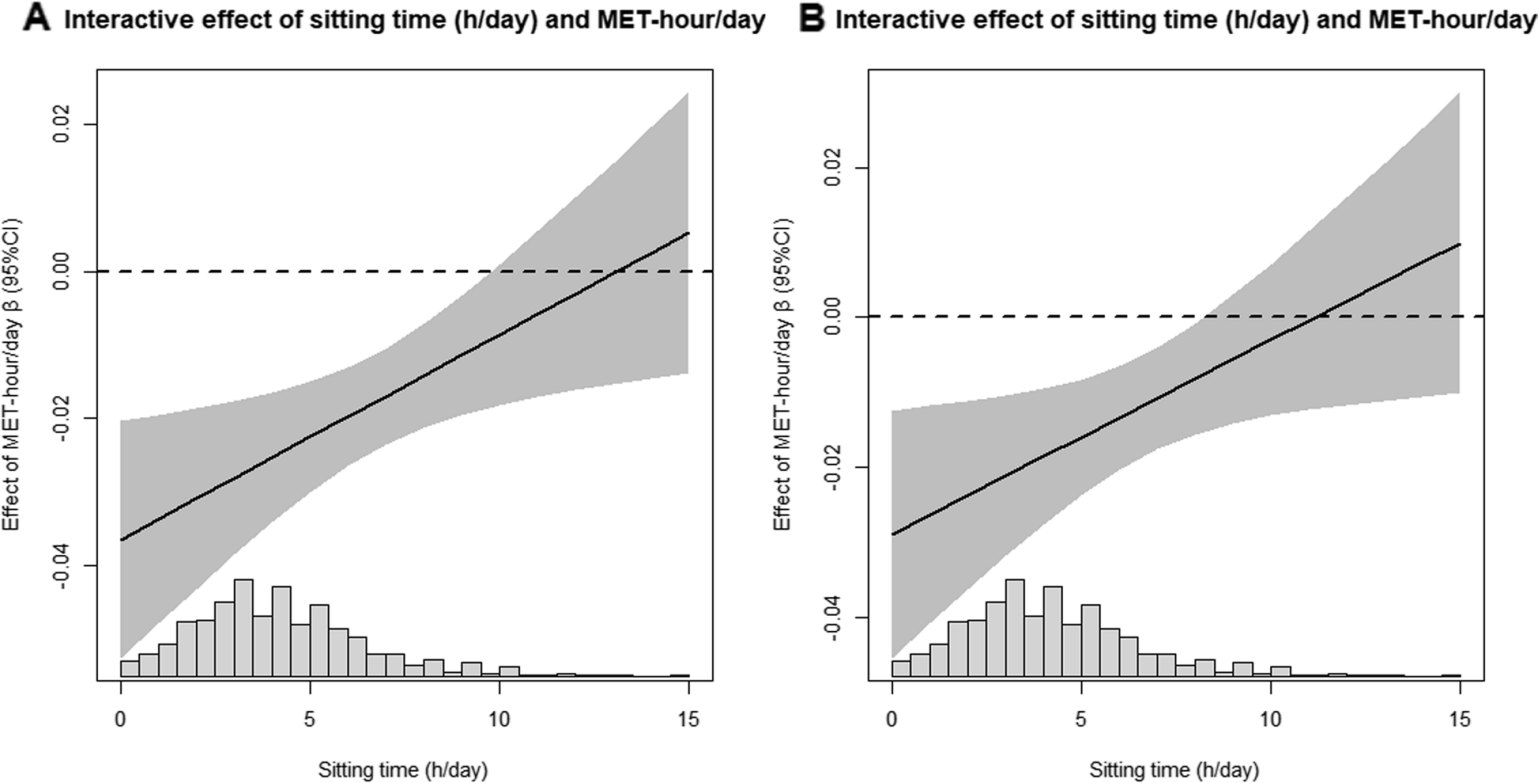
Interactive effect of physical activity (PA) (MET-h/day) and ST (h/day) on risk of frailty. **A** Model 1: Adjusted for physical activity level and sitting time. **B** Model 2: Adjusted for educational level, living status, marital status, economic income, drinking status, comorbidity and polypharmacy. The black lines and gray areas represented the estimated effect and 95% CI of physical activity on risk of frailty along with changed values of sitting time (h/day)

To explore the interactive effect of PA and ST on frailty, we adopted a generalised linear model to estimate the effect of MET (h/day) of PA on risk of frailty as a function of sitting time (h/day). After adjusting for potential confounding variables in model 2, the results showed that the estimated effect of PA on frailty risk varied with increased sedentary time. The protective effect of MET (h/day) of PA on risk of frailty decreased with increased ST (h/day), as shown in Fig. 2.

## Discussion

The current study examined the independent and interactive effects of PA and sedentary behaviour with frailty in Chinese community-dwelling older adults. Our findings suggest that dose-response relationship between low PA, high levels of ST with an increased risk of frailty. In addition, the results indicate that the protective effects of PA-MET (h/day) on frailty were weaken by increasing ST with a significant interaction effect (*P* < 0.001).

There has been an increasing number of studies on the dose-response relationship between PA, ST and frailty [28–30], however, the dose-response curve model is not always well explored [31]. Using a restricted cubic spline model, we found a non-linear dose-response relationship between total PA and frailty. The incidence of frailty decreased by 7.2% for each 1 MET-h /day increase in PA. The risk of frailty decreased with increasing PA, but the curve flattened out beyond 5 MET h/d. The negative association between PA and frailty in the present study was consistent with previous studies, and the magnitude of the association was comparable. In García-Esquinas E’s study, every 1MET-h /week increase in self-reported PA, the incidence of frailty decreased by 6% [32].

There is a linear dose-response relationship between ST and frailty. For each additional h of sedentary time, the risk of frailty increased by 11.4%. When the sedentary time was less than 4 h/d, the slope of frailty risk changed little, and when it is more than 8 h/d, the slope shows a very sharp upward trend. There are few studies on the dose-response relationship between sedentary time and frailty. A systematic review showed that participants with the highest sedentary had an odds or hazard ratio of 1.47 to 11.88 for becoming frail at follow-up compared with individuals with the least sedentary time [33, 34]. DA Silva Coqueiro reported that 7-hours of daily self-reported SB was the best cut-off point for distinguishing frail individuals [35]. However, this cut-off point is lower compared to objectively measured SB [16].

Interestingly, the negative association between PA and frailty was reduced by increasing ST, with a significant interaction (*P* < 0.001). This means that sedentary time reduces the protective effect of PA on frailty. In addition, in the joint analysis, light PA with the longest ST (> 8 h/day) was associated with a 8-fold increased risk of frailty in older adults compared to vigorous PA with the least ST (<4 h/day). Likewise, the ‘light physical activity & <4h sitting time’ group had significantly lower frailty compared to the ‘vigorous physical activity & >8 h sitting time’ group. This indicates that physical activity and sedentary time have an offsetting effect in addition to the additive effect. Supporting our results are the findings of Asier Mañas et al. In the Toledo Study of Healthy Aging, the authors used the Johnson-Neyman technique to find that 27.25 minutes of MVPA per day offset the adverse frailty effects of ST [36]. Furthermore, using isotemporal substitution and cross-lagged panel models, they found that replacing ST with MVPA was associated with a theoretically positive effect on frailty [37]. It was also confirmed that participants who spend less time on MVPA at baseline was more likely to increase their frailty score at follow up [38, 39].

There is limited understanding of the biological mechanisms underlying the interaction of PA, sedentary behaviour and frailty. Frailty is the result of an interaction between the ageing process and chronic diseases and is associated with the activation of inflammatory pathways. Physical activity results in reduced age-related oxidative damage, chronic inflammation and insulin sensitivity [40]. Sedentary behaviors produce cardiometabolic markers such as insulin resistance factors and increase inflammatory factors and incapacity [41]. In addition, prolonged ST may cause exercise resistance and reduce the benefits of PA. These two distinct behavioral aspects, when combined, may exacerbate physiological changes caused by the ageing process itself, leading to reductions in total energy expenditure, maximal oxygen consumption and resting metabolic rate.

Our study suggests that public health messages to older people living in communities should clarify the difference and interaction between reducing sedentary behaviors and increasing physical activity. The World Health Organization in its 2020 Global Guidelines on PA and Sedentary Behaviour recommends limiting sedentary behaviour and replacing it with healthy PA to improve health, especially for individuals with long-term conditions [42]. The optimal combined dose of PA and sedentary time in the frailty population remains unknown. Therefore, it is important to effectively implement population-based prevention measures before the onset of functional decline in the elderly. Community health care workers should strengthen social and environmental support for reducing sedentary time among older adults, including implementing strategies to improve PA facilities and modifying public and private spaces to reduce sedentary behaviour [43]. In addition, it is important to provide social support to older adults, including exercise with peers, fun forms of PA and friendly social interactions, as it is difficult to maintain PA because of the impact of COVID-19 and the deterioration of physical function [44].

One of the strengths of our study is that the dose-response association between PA or ST and frailty was evaluated by restricted cubic splines, which has not been carried out in other studies in the Chinese population. In addition, this study is one of the few studies that investigate the interaction effect of PA and ST on frailty in the context of regular COVID-19 epidemic prevention and control in China. Third, most studies have assessed frailty using a frailty phenotype. Although this tool provides information about changes in physical vulnerability, it may not fully capture the complexities of vulnerability and ageing. Our study measured frailty through the FI to further understand the effects of PA and ST on frailty.

Some limitations in the current study should be taken into account. First, only cohort baseline survey data were used in this study, and a causal relationship between PA, ST and frailty could not be established because of the cross-sectional study design. Second, demographic information and lifestyle characteristics, including PA and ST, were collected through questionnaires, so recall bias may be unavoidable. Third, while we present many potential confounding factors, it is likely that some remaining confounding factors may have influenced the estimates.

## Conclusions

In conclusion, this study suggests that non-linear and linear dose-respons relationship exist between PA, ST and frailty respectively. In addition, excess ST may counteract the beneficial effects of PA on the frailty. The lowest PA with the longest SB was associated with a 8-fold increased risk of frailty compared to highest PA with the least SB. The ‘light physical activity & <4h sitting time’ group had significantly lower frailty compared to the ‘vigorous physical activity & >8 h sitting time’ group. It is suggested that regular PA and reduction of ST play an important role in preventing frailty. In addition, interventions that focus on reducing excess ST may be effective strategies to reduce the risk of frailty and should be taken seriously by public health authorities, especially in the context of regular epidemic prevention and control in China.

## Supplementary Information

**Additional file 1: Figure S1.** The flowchart of participant selection. **Table S1.** Health Variables and Cut-points for the Frailty Index.

## Data Availability

All data generated or analysed during this study are included in this published article [and its supplementary information files].

## Abbreviations

PA: Physical activity
SB: Sedentary behavior
FI: Frailty index
OR: Odds ratio
CI: Confidence interval
