# Supplementary material for: Examining the independent and interactive association of physical activity and sedentary behaviour with frailty in Chinese community-dwelling older adults
Source: BMC Public Health. 2022 Jul 26;22:1414. doi: 10.1186/s12889-022-13842-1 (PMC9313602; doi:10.1186/s12889-022-13842-1)
Supplement: Supplementary file 1 — Additional file 1: Figure S1. The flowchart of participant selection. Table S1. Health Variables and Cut-points for the Frailty Index. [file 12889_2022_13842_MOESM1_ESM.docx]

A total of 1508 participants over the age of 60 were recruited from Community Health Service Center of Fujian Province

Participants were excluded if they have extreme SB and PA values (n = 14)

Participants were further excluded with incomplete information on frailty index: (Grip Strength, Balance, Fatigue) (n = 29) and potential confounders (n = 7)

1458 participants were included in the analysis

**Figure S1** The flowchart of participant selection

**Table S1 Health Variables and Cut-points for the Frailty Index**

| **List of 40 Variables included in the frailty index** | **Cut Point** |
| --- | --- |
| Help Bathing | Yes = 1, No = 0 |
| Help Dressing | Yes = 1, No = 0 |
| Help Using Toilet | Yes = 1, No = 0 |
| Help Eating | Yes = 1, No = 0 |
| Help Shopping | Yes = 1, No = 0 |
| Help with meal Preparations | Yes = 1, No = 0 |
| Help with Housework | Yes = 1, No = 0 |
| Help taking Medication | Yes = 1, No = 0 |
| Help with Finances | Yes = 1, No = 0 |
| Help Grooming | Yes = 1, No = 0 |
| Help lifting 10 lbs | Yes = 1, No = 0 |
| Help getting in/out of Chair | Yes = 1, No = 0 |
| Help Walking around house | Yes = 1, No = 0 |
| Help up/down Stairs | Yes = 1, No = 0 |
| Self Rating of Health | Poor = 1, Fair = 0.5, Good = 0 |
| High blood pressure | Yes = 1, No = 0 |
| Diabetes | Yes = 1, No = 0 |
| Cancer | Yes = 1, No = 0 |
| Stroke | Yes = 1, No = 0 |
| CHF | Yes = 1, No = 0 |
| Chronic Lung Disease | Yes = 1, No = 0 |
| Heart attract | Yes = 1, No = 0 |
| Coronary heart disease | Yes = 1, No = 0 |
| Number of Medications | ≥5 =1，<5 =0 |
| Nutrition | MNA-SF <12=1, ≥12=0 |
| Mobility | Yes = 1, No = 0 |
| Vision problem | Poor = 1, Fair = 0.5, Good = 0 |
| Hearing problem | Poor = 1, Fair = 0.5, Good = 0 |
| Fatigue | Yes = 1, No = 0 |
| Balance | TUG >20s=1, 10~20s=0.5, <10s=0 |
| Urinary incontinence | Most of time = 1, Some time = 0.5, Rarely = 0 |
| A history of falls | More than 2 times or serious injury = 1,1 time =0.5, No=0 |
| Anxiety | GAD-7≥14=1, 5~13=0.5, ≤4=0 |
| Depression | GDS-4≥2 =1, 1point=0.5, 0point=0 |
| Sleep | AIS≥6=1, 4~5 point=0.5, <4 point=0 |
| Chronic pain | Yes= 1, No= 0 |
| Cognition | Minicog≤2=1, =3=0.5, >3=0 |
| Grip Strength | Men  BMI <24 🡪 <29  BMI 24.1–26 🡪 <30  BMI 26.1–28 🡪 <30  BMI >28 🡪 <32  Women  BMI <23 🡪 <17  BMI 23.1–26🡪 <17.3  BMI 26.1–29 🡪 <18  BMI >29 🡪 <21 |
| walking speed | Walking time / 15 feet  Men  Height <173 cm (68.1 in) 🡪 >7 seconds  Height >173 cm (68.1 in) 🡪 >6 seconds  Women  Height <159 cm (62.6 in) 🡪 >7 seconds  Height >159 cm (62.6 in) 🡪 >6 seconds |
| Social support | SSRS<11point=1, ≥11=0 |

Note : TUG=time up and go test, GAD-7=Generalized Anxiexy Disorde-7, GDS-4=The Geriatric Depression Scale-4, AIS=Athens Insomnia Scale, Minicog=Minicog simple cognitive scale, SSRS=Social Support Rating Scale
